# Enhanced Permeability and Binding Activity of Isobutylene‐Grafted Peptides

**DOI:** 10.1002/cbic.201700586

**Published:** 2017-11-22

**Authors:** Shuang Sun, Ismael Compañón, Nuria Martínez‐Sáez, João D. Seixas, Omar Boutureira, Francisco Corzana, Gonçalo J. L. Bernardes

**Affiliations:** ^1^ Department of Chemistry University of Cambridge Lensfield Road Cambridge CB2 1EW UK; ^2^ Departamento de Química Centro de Investigación en Síntesis Química Universidad de La Rioja Madre de Dios, 53 26006 Logroño Spain; ^3^ Instituto de Medicina Molecular Faculdade de Medicina Universidade de Lisboa Avenida Professor Egas Moniz 1649-028 Lisboa Portugal

**Keywords:** cyclic peptides, isobutylene, macrocyclization, peptides, stapling

## Abstract

We present a new peptide‐macrocyclization strategy with an isobutylene graft. The reaction is mild and proceeds rapidly and efficiently both for linear and cyclic peptides. The resulting isobutylene‐grafted peptides possess improved passive membrane permeability due to the shielding of the polar backbone of the amides, as demonstrated by NMR spectroscopy and molecular dynamics simulations. The isobutylene‐stapled structures are fully stable in human plasma and in the presence of glutathione. This strategy can be applied to bioactive cyclic peptides such as somatostatin. Importantly, we found that structural preorganization forced by the isobutylene graft leads to a significant improvement in binding. The combined advantages of directness, selectivity, and smallness could allow application to peptide macrocyclization based on this attachment of the isobutylene graft.

Intramolecular side‐chain‐to‐side‐chain crosslinking, commonly termed “stapling” or “macrocyclization”,^1^ is an important technology in the development of bioactive peptide‐based therapeutics. Linear peptides are often easily degraded by proteases, and possess low binding affinity and cell permeability.^2^ Macrocyclization has evolved as a promising approach to tackling these problems. The cyclized structure often shows enhanced biophysical properties, cellular uptake, and binding affinity of peptides while maintaining high specificity for its biological targets.^3^ Over the past decades, the chemical toolbox available for macrocyclization has expanded greatly, and now includes disulfide bond formation,^4^ lactam formation,^5^ ring‐closing metathesis,^1^, ^6^ and cycloaddition.^7^ Proteinogenic cysteine has attracted significant interest as a convenient handle for stapling, owing to the high nucleophilicity of the thiolate and its unique reactivity.3a Chemical approaches for cysteine stapling include S‐alkylation,^8^ S_N_‐arylation,^9^ tetrazine stapling,^10^ and radical thiolene^11^ reactions. Among the cysteine‐stapling methods, S‐alkylation is the most flexible approach, as a wide range of bis‐thiol‐reactive linkers is commercially available. The first investigation of thiol‐reactive linkers by using 1,3,5‐tris(bromomethyl)benzene was reported in 1985,^12^ followed by that of its bis‐reactive analogues, 1,2‐ and 1,3‐bis(bromomethyl)benzene.^13^ In recent years, this strategy has also been successfully applied in peptide drug development.^14^ Despite the many tools available for peptide macrocyclization, discovering suitable grafts that yield membrane‐permeable cyclic peptides with enhanced binding affinities remains a great challenge.

Cysteine residues are easily incorporated into the peptide sequence through solid‐phase peptide synthesis. This facile incorporation is an important advantage over other stapling approaches based on nonproteinogenic amino acids. One challenge associated with cysteine macrocyclization strategies is potential oxidation to form disulfides. Thus, an efficient strategy for cysteine stapling should, in principle, be compatible with the presence of reducing agents in a mild, one‐pot reaction. Concurrently, the graft should be both small and biologically inert. Here, we described a new cysteine crosslinking strategy that allows the biocompatible and chemoselective installation of a small, rigid isobutylene graft (Scheme 1).

**Scheme 1:**
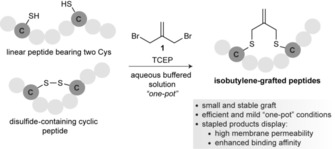
Schematic representation of the macrocyclization of peptides with cysteine residues by using bis‐electrophilic isobutylene.

Our investigation commenced with a linear pentamer model peptide CAAAC (peptide **I**, Scheme 2), which had cysteines at the *i* and *i+4* positions, prepared by Fmoc‐based solid‐phase peptide synthesis (SPPS). Tris(2‐carboxyethyl) phosphine (TCEP) was added to prevent formation of the disulfide, followed by the addition of stapling reagent **1**; this afforded exclusively the stapled peptide CAAAC **I′** in 71 % yield. The same procedure was then applied successfully to other hepta‐ and octamer linear peptides with different sequence compositions that containing cysteines at the *i*,*i*+6 or *i*,*i*+7 positions (Scheme 2). In each case, the linear peptides afforded the desired cyclic derivatives in high conversion and yield at room temperature. Finally, because the double bond of the isobutylene graft could potentially be used as a handle for further conjugation, we evaluated its reactivity under thiolene and inverse‐electron‐demand Diels–Alder conditions. We found that, under the conditions tested, the alkene did not act as a partner for either thiolene or inverse electron demand Diels–Alder (data not shown). This is a significant difference compared to the method described by Dawson and co‐workers,8c in which the introduced moiety can be used for further conjugation. On the other hand, the isobutylene scaffold is more flexible than the bis(bromomethyl)benzene platform used in the CLIPS strategy;8b in some cases this could be a competitive advantage for selecting the bioactive conformation.

**Scheme 2:**
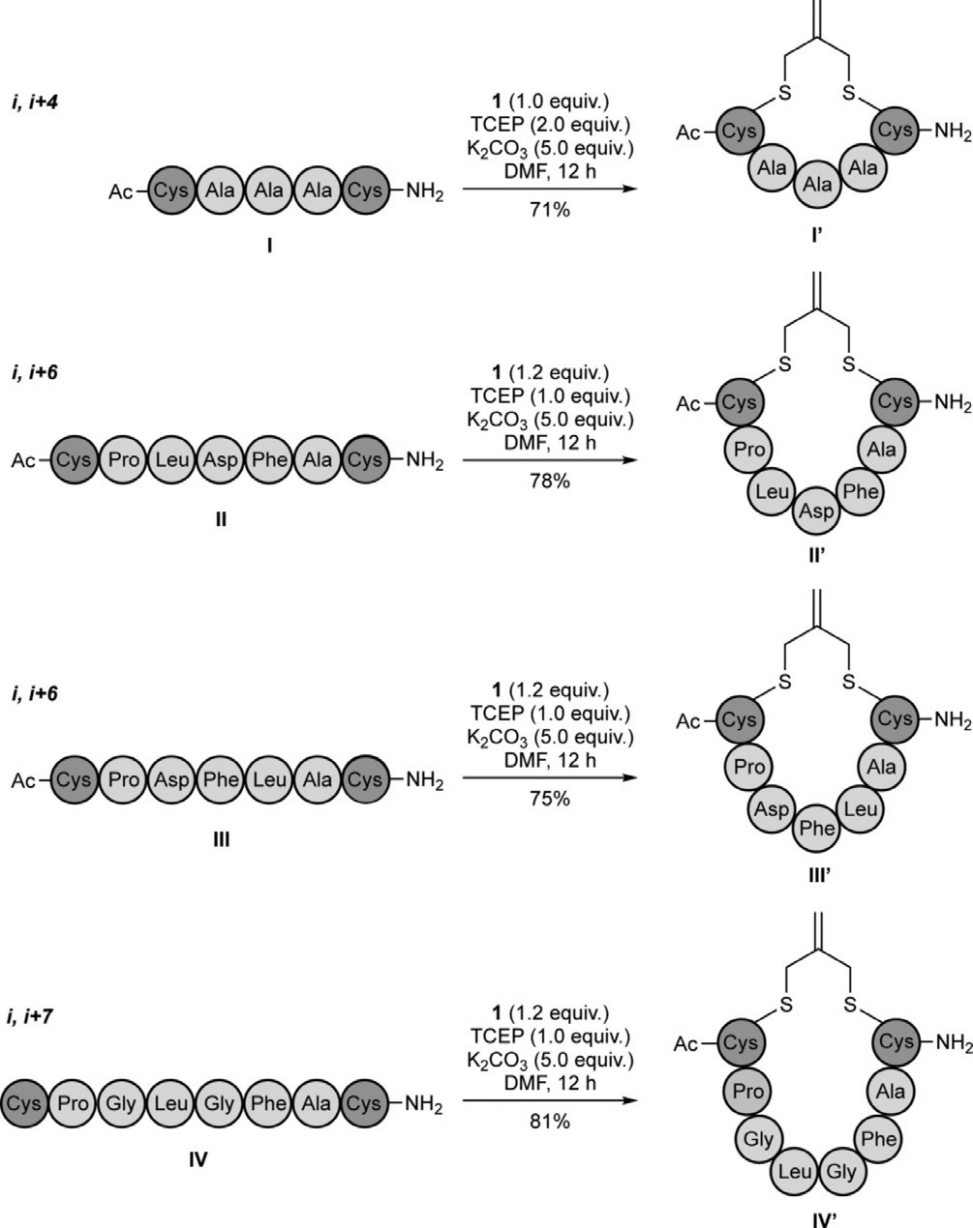
Macrocyclization of linear peptides with cysteines at the (*i*,*i*+4), (*i*,*i*+6) and (*i*,*i*+7) positions.

Next, we determined the structural changes induced by the incorporation of the isobutylene staple into the large (*i*,*i+7*) peptide **IV** by combining NMR spectroscopy and molecular dynamics (MD) simulations. The 2D ROESY spectra showed substantial differences between the unstapled and stapled peptide in terms of their conformational preferences (Figure 1 A and B, Supporting information). Clear medium‐sized NH–NH NOE crosspeaks, which are characteristic of a predominantly folded conformation in solution, were observed for stapled peptide **IV′**; the absence of these NH–NH NOE crosspeaks for unstapled peptide **IV** is in agreement with an extended disposition of the backbone.^15^ To obtain an experimentally derived conformational ensemble of compounds **IV** and **IV′**, 20‐ns MD simulations with time‐averaged restraints (MD‐tar)^16^ were carried out in explicit water with the key experimental distances included as restraints. The MD‐tar simulations were performed by using the AMBER 16^17^ package with parm14SB and GAFF force fields.^18^ The good agreement found between the experimental and theoretical distances validates the outcome of the MD‐tar calculations (see the Supporting Information). According to these calculations, peptide **IV** is reasonably flexible in solution, presenting a random‐coil distribution for its backbone. Conversely, stapled peptide **IV′** is rather rigid, showing a folded backbone held by the isobutylene staple (Figure 1 C and D, Supporting Information).

**Figure 1:**
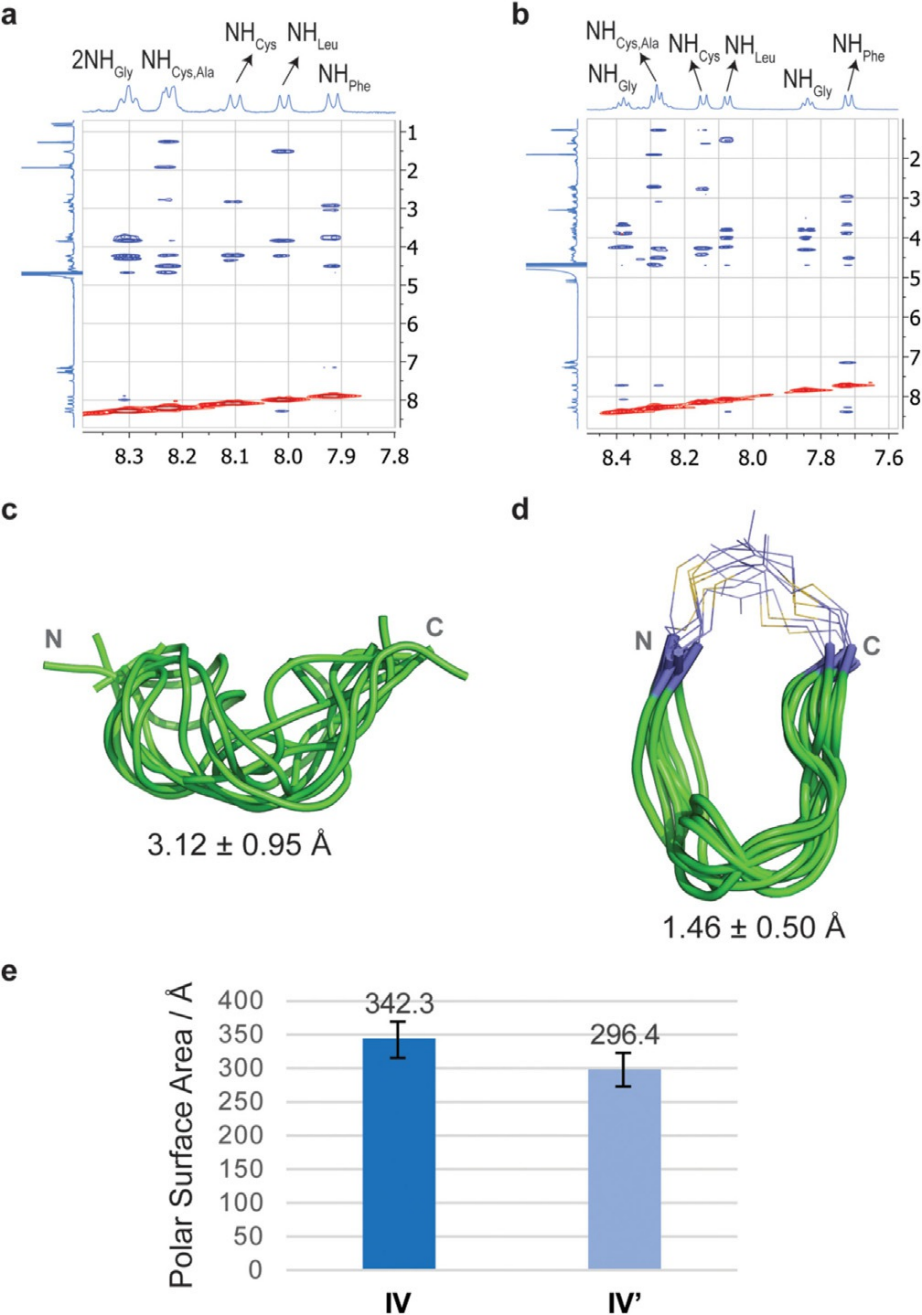
Conformation analysis of stapled and unstapled peptides in solution. Sections of the 500 ms ROESY spectra (400 MHz) of peptides A) **IV** and B) **IV′** in H_2_O/D_2_O (9:1) at pH 6.5 and 20 °C, showing amide–aliphatic crosspeaks. Structural ensembles obtained for C) peptide **IV** and D) stapled peptide **IV′** through 20‐ns MD‐tar simulations. The backbone is shown in green, and the carbon atoms of isobutylene moiety are in purple. The numbers indicate the rmsd for heavy‐atom superimposition of the backbone with respect to the average structure. E) PSA estimated for peptides **IV** and **IV′** through the MD‐tar simulations.

We then estimated the solvent‐exposed polar surface area (PSA) of peptides **IV** and **IV′** through the MD simulations. The stapled peptide **IV′** displayed around 15 % less PSA than the unstapled variant **IV**; this suggests that the folded conformation forced by the isobutylene fragment promotes shielding of the polar backbone amides (Figure S26). We then decided to determine the passive membrane permeability of peptides **II**–**IV** and their stapled variants experimentally, a key feature for the development of peptide‐based therapeutics (Table 1).^19^ All the isobutylene‐grafted peptides had a significant improvement in permeability compared to their linear forms, particularly compound **IV′**. For all stapled derivatives, we observed values of −log *P*_e_<5.0; this is indicative of highly passively permeable compounds.^20^ These data highlight the practicability of the method for developing bioactive peptides with favorable properties.

**Table 1:** The parallel artificial membrane permeability assay (PAMPA).^[a]^

Compound	Permeability	−log *P*_e_	Compound	Permeability	−log *P*_e_
	[nm s^−1^]			[nm s^−1^]	
**II**	7.6	5.12	**II′**	12	4.96
**III**	<0.01		**III′**	10	4.99
**IV**	6.0	5.22	**IV′**	13	4.90

[a] Permeability was measured at pH 7.4 and at room temperature, the value is reported as an average of quadruplicates.

The feasibility of our stapling approach in aqueous solution, and the impact of the isobutylene scaffold on bioactivity were evaluated further with somatostatin. This peptide inhibits the release of growth hormone, insulin and glucagon, and possesses a disulfide bond between two cysteines at the *i* and *i*+11 positions.^21^ Unlike the disulfide bond, which is sensitive to the biological environment, especially in the presence of biological thiols, the stapled form with the isobutylene graft can improve pharmacokinetics and binding affinity. The reaction was conducted in water with 10 % DMF as co‐solvent, and afforded the stapled somatostatin quantitatively (Figure 2 A). This is possible because our method is compatible with TCEP and can be performed in one pot. Notably, the stapled somatostatin was fully stable in the presence of glutathione and human plasma at 37 °C for 48 h (Figures S20 and S21). The affinity of the peptide for the somatostatin receptor type 2 (SSTR2) was experimentally determined by tryptophan fluorescence spectroscopy.^22^ As shown in Figure 2 B, the fluorescence emission peak of pure SSTR2 solution was at 328 nm. After increasing the concentration of either native or stapled somatostatin, the emission peak of both solutions shifted to 338 nm, with a decrease in intensity. Subsequent addition of somatostatin did not cause any shift in either peak, thus indicating the saturation of SSTR2. The minimum concentration of the somatostatin surrogate required to achieve saturation was 3.5 μm; in contrast, at least 5.5 μm of the native somatostatin was needed. These data suggest that the isobutylene‐grafted somatostatin has a higher binding affinity for SSTR2. This improvement in binding activity represents a considerable advantage of the incorporation of the isobutylene graft when compared to other three‐carbon grafts, such as the recently reported methylene thioacetal, which led to a decrease in affinity for SSTR2.8e Our 0.5‐μs unrestrained MD simulations performed on both derivatives indicated that the stapled somatostatin is more rigid in solution (Figure 2 C). Although the circular dichroism (CD) spectra of somatostatin and the corresponding stapled peptide (Figure 2 D and the Supporting Information) are rather similar, the peak at 225 nm found in somatostatin might be indicative of the presence of a higher degree of polyproline (PPII) conformation for this peptide.^23^ MD simulations showed the S−S distance in the isobutylene scaffold to be around 4.2±0.4 Å—larger than the conventional S−S disulfide bond length (ca. 2.0 Å) and the S−S distance in methylene thioacetals (close to 3.0 Å);8e hypothetically this would allow the required degree of flexibility to adopt a bioactive conformation. The restrained peptide flexibility and structural preorganization within the backbone, favored by the formation of intramolecular hydrogen bonds, might reduce the entropy cost of receptor binding that limits the conformational ensemble and, ultimately, increase the binding affinity compared to those of disulfide cyclized analogues.^24^

**Figure 2:**
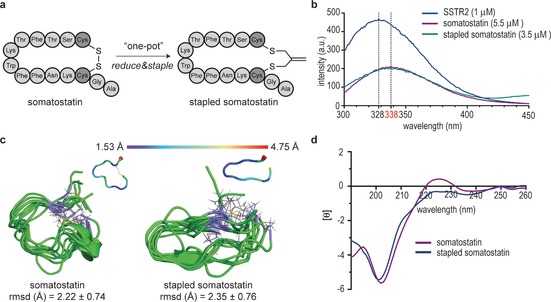
Stapling as well as structural and biological evaluation of somatostatin. A) Schematic representation of the stapling of somatostatin; B) Tryptophan fluorescence spectroscopy of somatostatin. Blue: 1 μm SSTR2 in buffer; purple: 5.5 μm native somatostatin and 1 μm SSTR2 in buffer; green: 3.5 μm stapled somatostatin and 1 μm SSTR2 in buffer. C) Structural ensembles obtained for native and stapled somatostatin through 0.5‐μs unrestrained MD simulations. The atomic fluctuation (Cα) analysis of both peptides is also shown. The data correspond to the average structure of both molecules throughout the simulations. The backbone is shown in green, and carbon atoms of cysteine isobutylene residues are in purple. The numbers indicate the rmsd for heavy‐atom superimposition of the backbone with respect to the average structure. D) CD spectra of native and stapled somatostatin.

In conclusion, we have demonstrated a robust cysteine macrocyclization and stapling strategy in which an isobutylene graft is introduced in a one‐pot (with TCEP), biocompatible manner. This method was applied to several linear peptides of various sequence composition and a bioactive disulfide cyclic peptide. The shielding of the polar backbone of the amides promoted by the isobutylene graft led to highly membrane‐permeable peptide macrocycles. Enhanced binding activity, resulting from limited flexibility and structural preorganization of the peptide backbone, was also observed. We believe that this access to such a “small” site‐selectively introduced isobutylene, which is less disruptive than many current bulky grafts, as demonstrated here for linear and cyclic peptides, is likely to find significant use for the peptide drug discovery community by allowing access to structures with improved properties.

## Experimental Section

**General procedure for peptide stapling with isobutylene**: The linear peptide (0.02 mmol) was dissolved in DMF (10 mL). K_2_CO_3_ (0.10 mmol) and tris(2‐carboxyethyl)phosphine (TCEP; 0.02 mmol) were then added. The solution was stirred for 1 h at room temperature. 3‐Bromo‐2‐(bromomethyl)prop‐1‐ene (0.025 mmol) was then added, and the mixture was stirred for an additional 12 h. The crude peptide was purified by reversed‐phase HPLC to obtain the corresponding stapled derivative. In all cases the yield was ≥75 %.

## Conflict of interest

*S.S., N.M.S., O.B., F.C., and G.J.L.B. are listed as inventors in a patent application related to the work presented here*.

## Supporting information

As a service to our authors and readers, this journal provides supporting information supplied by the authors. Such materials are peer reviewed and may be re‐organized for online delivery, but are not copy‐edited or typeset. Technical support issues arising from supporting information (other than missing files) should be addressed to the authors.

Supplementary
