# Supplementary material for: Enhanced Permeability and Binding Activity of Isobutylene‐Grafted Peptides
Source: Chembiochem. 2017 Nov 22;19(1):48–52. doi: 10.1002/cbic.201700586 (PMC5813187; doi:10.1002/cbic.201700586)
Supplement: Supplementary file 1 — Supplementary [file CBIC-19-48-s001.pdf]

CHEMBIOCHEM

Supporting Information

Enhanced Permeability and Binding Activity of Isobutylene-Grafted Peptides

Shuang Sun,[a] Ismael Compañón,[b] Nuria Martínez-Sáez,[a] João D. Seixas,[c] Omar Boutureira,[a] Francisco Corzana,*[b] and Gonçalo J. L. Bernardes*[a, c]

cbic_201700586_sm_miscellaneous_information.pdf

Supporting Information

Enhanced permeability and binding activity of isobutylene-grafted peptides

Table of Contents

General methods	S1
Synthesis and characterization of peptides	S6
Circular Dichroism spectroscopy	S15
Parallel Artificial Membrane Permeability Assay (PAMPA)	S15
Tryptophan fluorescence spectroscopy	S16
Stability test of Stapled Somatostatin	S16
ROESY spectra of peptides **IV** and **IV'**	S17
MD simulations data	S17
References	S19

General methods

Chromatography. Analytical thin-layer chromatography (TLC) was carried out on Merck silica gel 60 F_{254} plates, using UV at 254 nm or staining with ninhydrin for visualization. Column chromatography was performed with Material Harvest silica gel 60. Reverse-phase column chromatography was conducted with Varian Bond Elut® C18. The HPLC was conducted on Agilent 1100 Series fitted with G1322A degasser, G1311A pump, G1313A autosampler and G1315 DAD, with YMC-Pack Pro C18 column 120 Å S-5 µm 10 mm x 250 mm (product no. AS12S05-2510WT) for preparative scale. The eluent was solvent B, water with 0.1% trifluoroacetic acid (TFA), and C, acetonitrile with 0.1% trifluoroacetic acid, unless otherwise noted and gradients specific to the compound.

Characterization. ^{1}H NMR was recorded on Bruker 400-Avance III, DPX-400 or 500-DCH Cryoprobe as appropriate. ^{13}C NMR were recorded by the Department of Chemistry NMR service. Chemical shifts (ppm) were referenced to the residual proton signal of the solvent. High resolution mass spectra were obtained by the Department of Chemistry Mass spectrometry service with a Thermo Fischer LTQ Orbitrap Discovery and ionised by electrospray (ESI).

2D ROESY experiments. ROESY experiments on peptides **IV** and **IV'** were recorded on a Bruker Avance 400 spectrometer at 298 K and pH 5.6 in H_2O/D_2O (9:1). The experiments were conducted by using phase-sensitive ge-2D ROESY with WATERGATE for H_2O/D_2O (9:1) spectra. ROESY intensities were normalized with respect to the diagonal peak at zero mixing time. Distances involving NH protons were semi-quantitatively determined by integrating the volume of the corresponding cross-peaks. The number of scans used was 32 and the mixing time was 500 ms.

Circular Dichroism spectroscopy. Peptide solutions were prepared from aqueous peptide stock solutions of accurate molecular concentrations determined by NMR. The final concentration of the peptide samples was 1 mM in pure water. CD measurements were performed on an Aviv Model 410 spectrometer, which was routinely calibrated with (*1S*)-(+)-10-camphorsulfonic acid. Spectra were recorded at 298K with a 0.1 cm quartz cell over the wavelength range 250-189 nm at 50 $nm\cdot min^{-1}$, with a bandwidth of 1.0 nm, the response time of 1 s, resolution step width of 1 nm and sensitivity of 20-50 Mdeg. Each spectrum represents the average of 5 scans.

Parallel Artificial Membrane Permeability Assay (PAMPA). The PAMPA EvolutionTM instrument was used to determine permeability. In PAMPA, a sandwich is formed such that each composite well is divided into two chambers, separated by a 125 µm thick microfilter disc (0.45 µm pores), coated with Pion GIT-0 phospholipid mixture. The effective permeability, Pe, of each compound was measured at the customer-specified pHs in the donor compartment using low-binding, low UV Prisma buffer. The drug-free acceptor compartment was filled with acceptor sink buffer containing a scavenger at the start of the test. The proprietary scavenger mimics serum proteins and blood circulation, thus creating sink conditions.

In the default protocol the aqueous solutions of studied compounds are prepared by diluting and thoroughly mixing 3 μL of DMSO stock in 600 μL of Prisma HT buffer. Final concentration of organic solvent (DMSO) in aqueous buffer is ≤ 0.5% (v/v).

The reference solution is identical to the donor at time zero, so that any surface adsorption effects from the plastic is compensated. The PAMPA sandwich was assembled and allowed to incubate for ~15 hours. The solutions in the donor compartment were un-stirred within duration of the experiment. Thus, the thickness of the aqueous boundary layer expected to be about 1000 μm. The sandwich was then separated, and both the donor and receiver compartments were assayed for the amount of drug present by comparison with the UV spectrum obtained from reference standards. Mass balance was used to determine the amount of material remaining in the membrane filter and on the plastic (%R).

Ketoprofen, Verapmil and Propanolol were used as reference compounds.

Buffers preparation

pH of Prisma HT buffer was adjusted to the requested values using 1.0 M solution of NaOH. Actual pH the buffers was 7.40±0.05.

Stock solutions preparation

Sample powders pre-weighed in glass vials were brought to the room temperature at the day of the experiment. The samples were diluted with an organic solvent (DMSO) to prepare stock solutions at concentration ~10 mM. The stock solutions were further diluted in buffer at 7.40 producing the aqueous sample solutions at concentrations ~50 μM. The amount of DMSO in the resulting solution was <0.5% (v/v). The solutions were filtered prior assaying the samples.

Molecular dynamics (MD) simulations with time averaged restraints (MD-tar). The simulations on peptides **IV** and **IV'** were carried out with AMBER 16 package[S1] implemented with ff14SB [S2] and GAFF[S3] force fields. The parameters and charges for the unnatural amino acids were generated with the antechamber module of AMBER, using GAFF force field and AM1-BCC method for charges.[S4] Prior to MD-tar productive simulations, we performed an equilibration protocol consisting of an initial minimization of the water box of 5000 steps, followed by a 2500-step minimization of the whole system. Then, the TIP3P water[S5] box was heated at constant volume until 298 K, using a time constant for the heat bath coupling of 1 ps. The equilibration finished with 200 ps of MD simulation without restraints, at a constant pressure of 1 bar and turning on the Langevin temperature scaling with a collision frequency of 1 ps. Non-bonded interactions were cut-off at 8.0 Å and updated every 25 steps. Periodic boundary conditions and the Particle Mesh Ewald method[S6] were turned on in every step of the equilibration protocol to evaluate the long-range electrostatic forces, using a grid spacing of approximately 1 Å. The ROESY-derived distances were imposed as time-averaged constraint, applying an r^{-6} averaging. The equilibrium distance range was set to $r_{exp} - 0.2$ Å ≤ r_{exp} ≤ 0.2 Å. Trajectories were run at 298 K, with a decay constant of 2000 ps and a time step of 1 fs. The force constants rk_2 and rk_3 used in each case were 10 kcal·mol^{-1}·Å^{-2}. The overall simulation length was 20 ns. The coordinates were saved each 1 ps, obtaining MD trajectories of 20000

frames each. A convergence within the equilibrium distance range was obtained in the simulations.

Unrestrained (MD simulations on somatostatin and stapled somatostatin. Each peptide was immersed in a water box with a 10 Å buffer of TIP3P water molecules. A two-stage geometry optimization approach was performed. The first stage minimizes only the positions of solvent molecules and the second stage is an unrestrained minimization of all the atoms in the simulation cell. The systems were then gently heated by incrementing the temperature from 0 to 300 K under a constant pressure of 1 atm and periodic boundary conditions. Harmonic restraints of 30 $kcal \cdot mol^{-1}$ were applied to the solute, and the Andersen temperature-coupling scheme was used to control and equalize the temperature. The time step was kept at 1 fs during the heating stages, allowing potential inhomogeneities to self-adjust. Long-range electrostatic effects were modelled using the particle-mesh-Ewald method. An 8 Å cut-off was applied to Lennard-Jones and electrostatic interactions. Each system was equilibrated for 2 ns with a 2 fs time step at a constant volume and temperature of 300 K. Production trajectories were then run for additional 500 ns under the same simulation conditions.

Polar surface area (PSA) for glycopeptides IV and IV'. This parameter was obtained by MD-tar simulations with CPPTRAJ software,[S7] included in AMBER16, using the 'surf' flag and the mask :1-10@O,N.

Synthesis of peptides

Linear peptides

Rink Resin Amino Acid Loading. Rink Amide MBHA Resin (0.2 mmol) was placed in a peptide synthesis vessel and treated with 25% piperidine/DMF (1 mL/3 mL) and rocked gently for 1 h. The resin was washed with DMF (3 mL), DCM (3 mL), DMF (3 mL). Pre-mixed solution of Fmoc-protected amino acid (0.8 mmol, 4 equiv), OxymaPure (142 mg, 1.0 mmol, 5 equiv) and DIC (155 µL, 1.0 mmol, 5 equiv) dissolved in DMF (3 mL) was added to the resin. The contents were rocked gently for 2 h, then drained and the resin washed with DMF (3 mL), DCM (3 mL), DMF (3 mL). The Fmoc deprotection procedure was repeated followed by the coupling of the next amino acid in the sequence to synthesize the desired peptide.

Peptide acetylation. After the terminal peptide was linked, the Fmoc was deprotected with the same method. Then the resin was treated with pyridine/Ac_2O (2 mL/1 mL) and rocked gently for 1 h. Then the resin was washed with DMF (3 mL), DCM (3 mL), DMF (3 mL).

Peptide Cleavage. The resin-bound peptide was treated with TFA/H_2O/EDT/TIS (2.82 mL/75 µL/75 µL/30 µL) and stirred under a nitrogen atmosphere for 2 hours. Et_2O (3 mL) was added to precipitate the peptide **I**. The white precipitate was collected by vacuum filtration and the solids wash with additional Et_2O. The crude peptide was dried and purified by HPLC, conducted on Agilent 1100 Series fitted with G1322A degasser, G1311A pump, G1313A autosampler and G1315 DAD, with YMC-Pack Pro C18 column (120 Å S-5 µm 10 mm x 250 mm, product no. AS12S05-2510WT) for preparative scale, with a flow rate of 3 mL/min.

Peptides **II**-**IV** were synthesized following a similar protocol, employing a stepwise micro-wave assisted solid-phase peptide synthesis on a Liberty Blue synthesizer. In these cases, the peptides were purified by HPLC using a Phenomenex Luna C18(2) column (10 μ, 250 mm × 21.2 mm) and a dual absorbance detector, with a flow rate of 20 mL/min.

Stapled peptides

The linear peptide (0.02 mmol) was dissolved in 10 mL of DMF and K_2CO_3 (0.10 mmol) and tris(2-carboxyethyl)phosphine (TCEP, 0.02 mmol) were ~~then~~ added. The solution was stirred for 1 h at rt. 3-bromo-2-bromomethyl-1-propene (0.025 mmol) was then added and stirred for additional 12 h. The crude peptide was purified by reversed-phase HPLC to obtain the corresponding stapled derivative. In all cases the yield was ≥ 75%. Finally, they were purified by HPLC.

Peptide I

HPLC: Rt = 10.27 min (Grad: water 0.1% TFA/acetonitrile (95:5) → (38.3:61.7), 20 min, λ = 212 nm). HRMS (ESI+) m/z: calcd. for $C_{17}H_{28}N_6O_6S_2Na$ $[M+Na]^+$ 499.1404 found: 499.1392

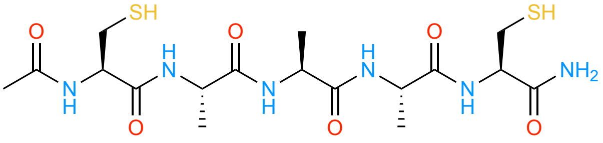

I

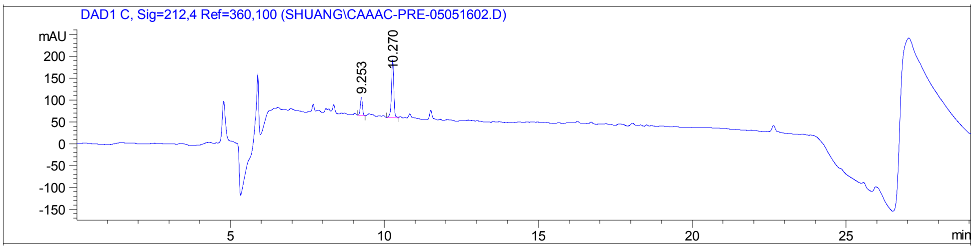

Figure S1. HPLC chromatogram of short peptide **I**. The signal at 9.25 min is disulfide form of **I**, and the signal at 10.27 min is linear form of **I**.

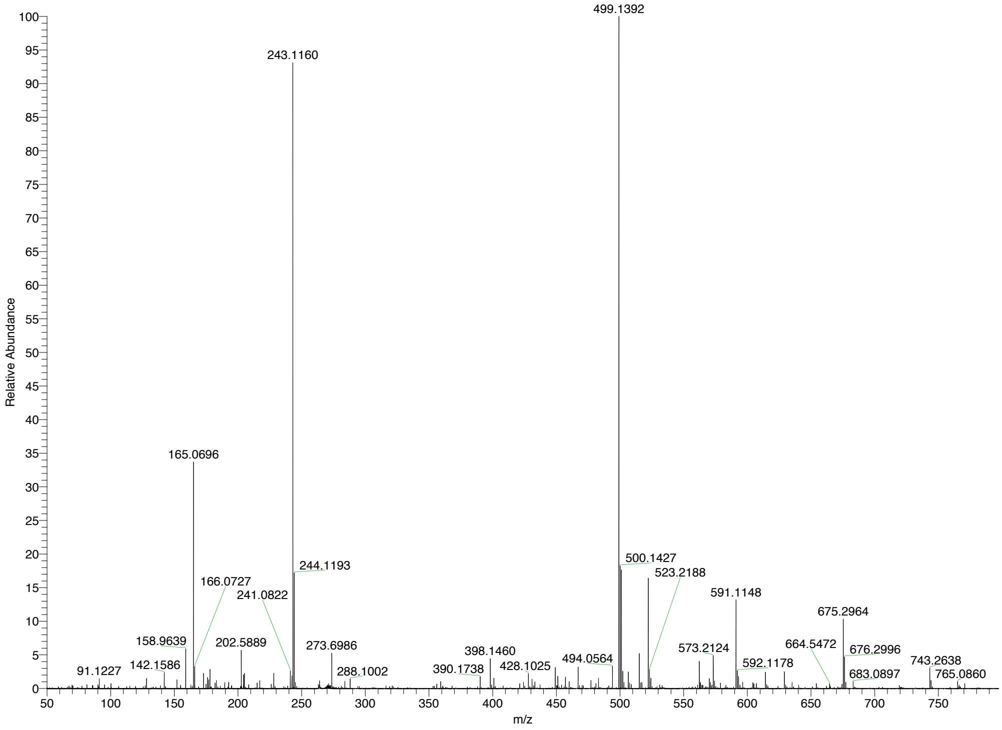

Figure S2. HRMS ESI+ of peptide **I**.

Peptide II

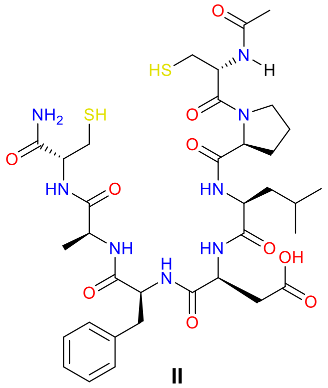

HPLC: Rt = 9.37 min (Grad: water 0.1% TFA/acetonitrile (70:30) → (63.5:36.5), 11 min, λ = 212 nm). HRMS ESI+ (m/z) calcd. for $C_{35}H_{53}N_8O_{10}S_2[M+H]^+$ 809.3321, found 809.3316.

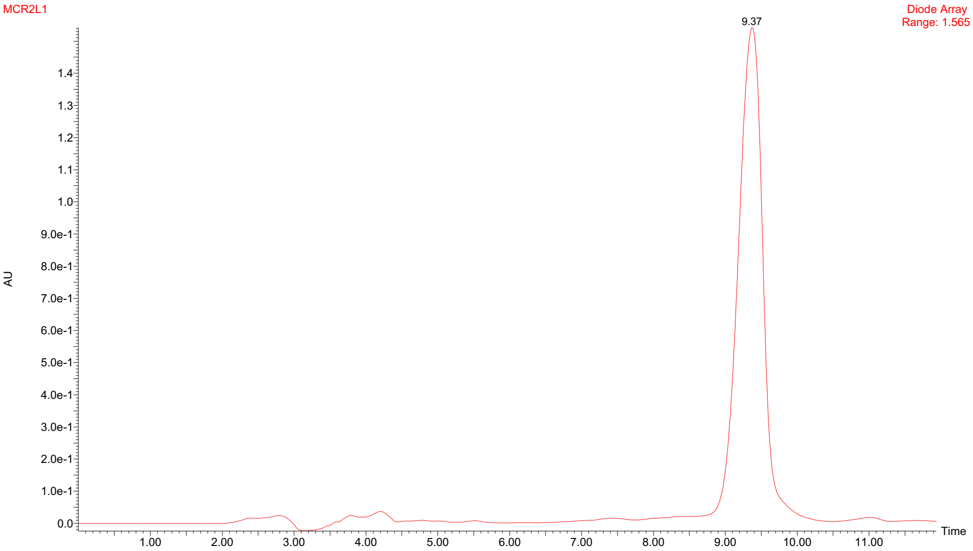

Figure S3. HPLC chromatogram of peptide **II**.

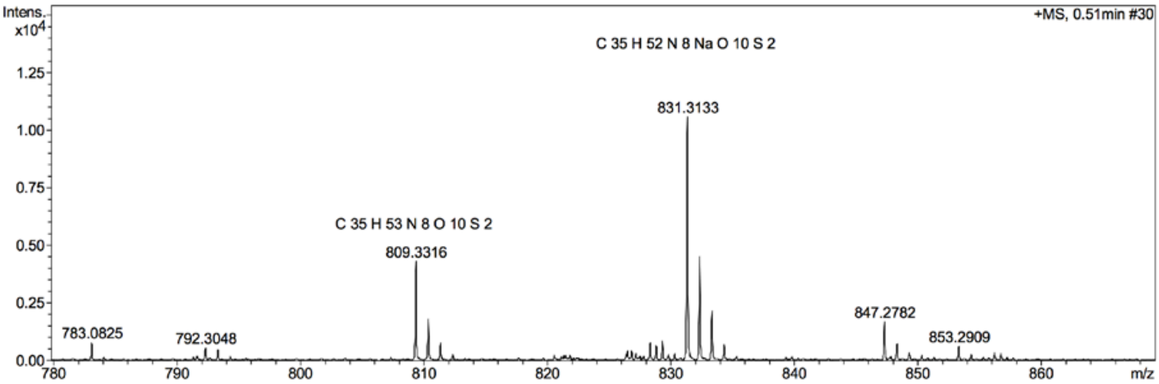

Formula	m/z	z	|err| [ppm]	Mean err [ppm]	mSigma
C 35 H 52 N 8 Na O 10 S 2	831.3140	1+	0.8	1.4	6.5
C 35 H 53 N 8 O 10 S 2	809.3321		0.6	0.9	11.1

Figure S4. HRMS ESI+ of peptide **II**.

Peptide III

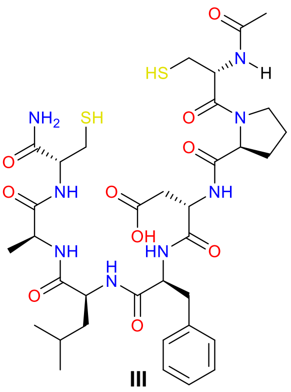

HPLC: Rt = 8.92 min (Grad: water 0.1% TFA/acetonitrile (70:30) → (63.5:36.5), 11 min, λ = 212 nm). HRMS ESI+ (m/z) calcd. for $C_{35}H_{53}N_8O_{10}S_2[M+H]^+$ 809.3321, found 809.3315.

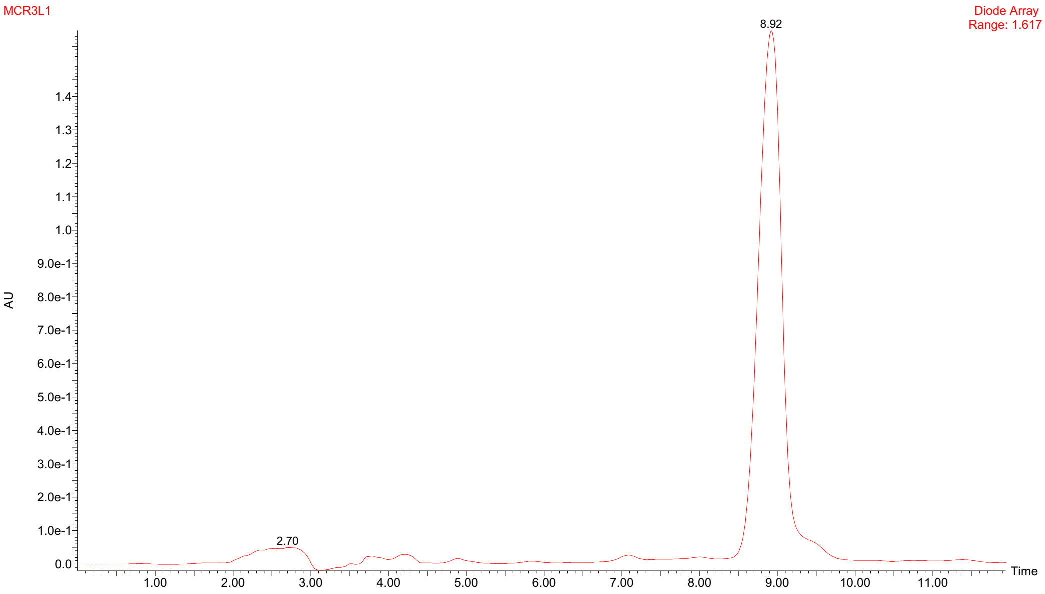

Figure S5. HPLC chromatogram of peptide **III**.

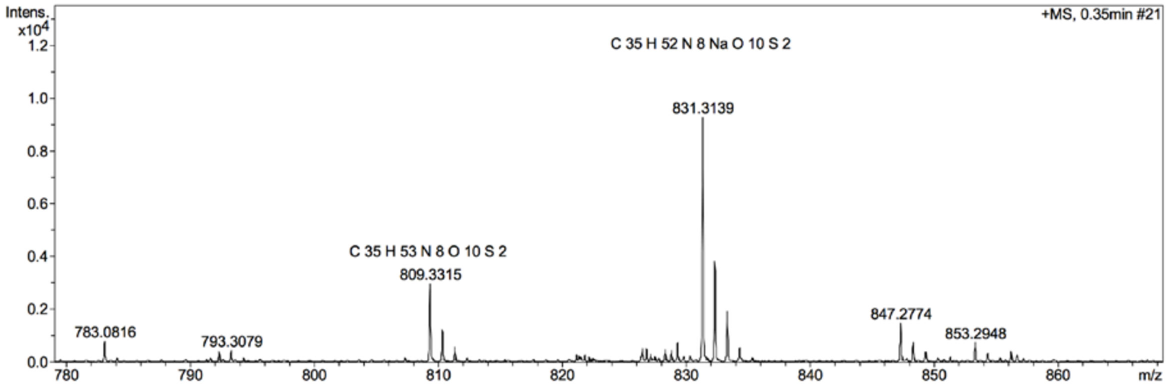

Formula	m/z	z	|err| [ppm]	Mean err [ppm]	mSigma
C 35 H 52 N 8 Na O 10 S 2	831.3140	1+	0.1	0.6	11.2
C 35 H 53 N 8 O 10 S 2	809.3321		0.7	-0.6	9.1

Figure S6. HRMS ESI+ of peptide **III**.

Peptide IV

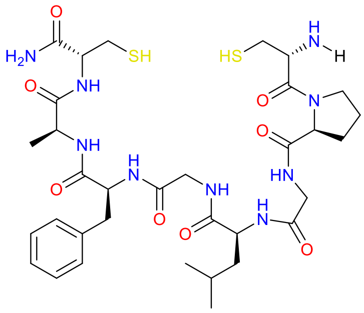

IV

HPLC: Rt = 7.07 min (Grad: water 0.1% TFA/acetonitrile (70:30) → (63.5:36.5), 11 min, λ = 212 nm). HRMS ESI+ (m/z) calcd. for $C_{35}H_{54}N_9O_9S_2[M+H]^+$ 808.3480, found 808.3462.

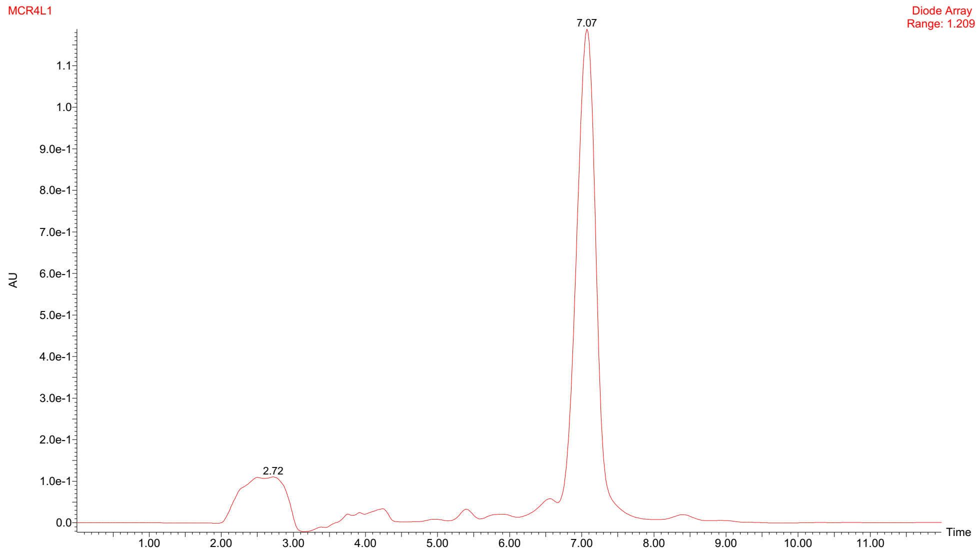

Figure S7. HPLC chromatogram of peptide **IV**.

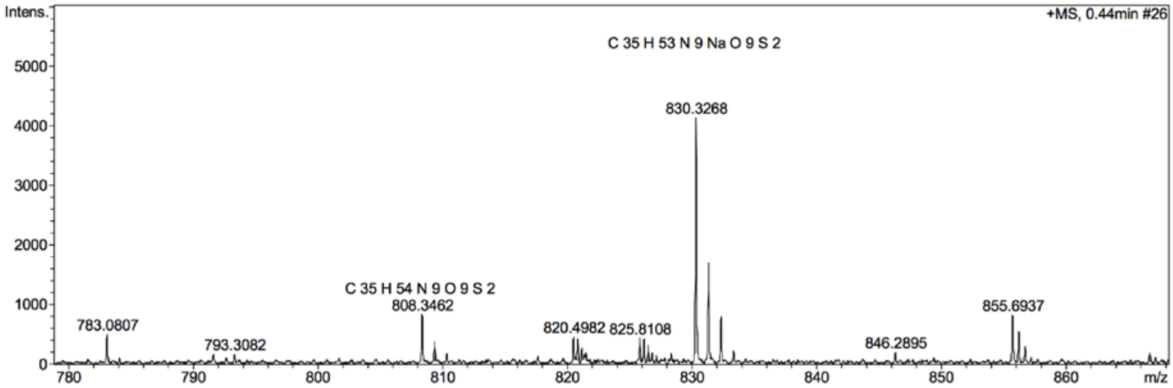

Formula	m/z	z	|err| [ppm]	Mean err [ppm]	mSigma
C 35 H 53 N 9 Na O 9 S 2	830.3300	1+	3.9	3.6	10.7
C 35 H 54 N 9 O 9 S 2	808.3480		2.2	2.7	30.1

Figure S8. HRMS ESI+ of peptide **IV**.

Peptide I'

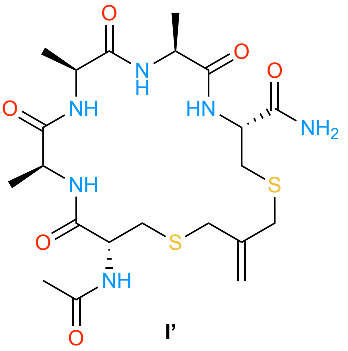

I'

HPLC: Rt = 13.05 min (Grad: water 0.1% TFA/acetonitrile (95:5) → (38.3:61.7), 20 min, λ = 212 nm). HRMS (ESI+) m/z: calcd. for $C_{21}H_{34}N_6O_6S_2Na$ $[M+Na]^+$ 553.1873 found: 553.1885.

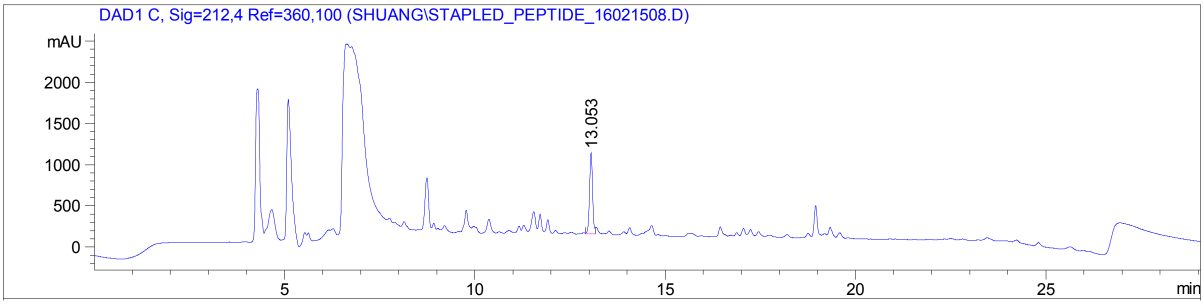

Figure S9. HPLC chromatogram of peptide **I'**.

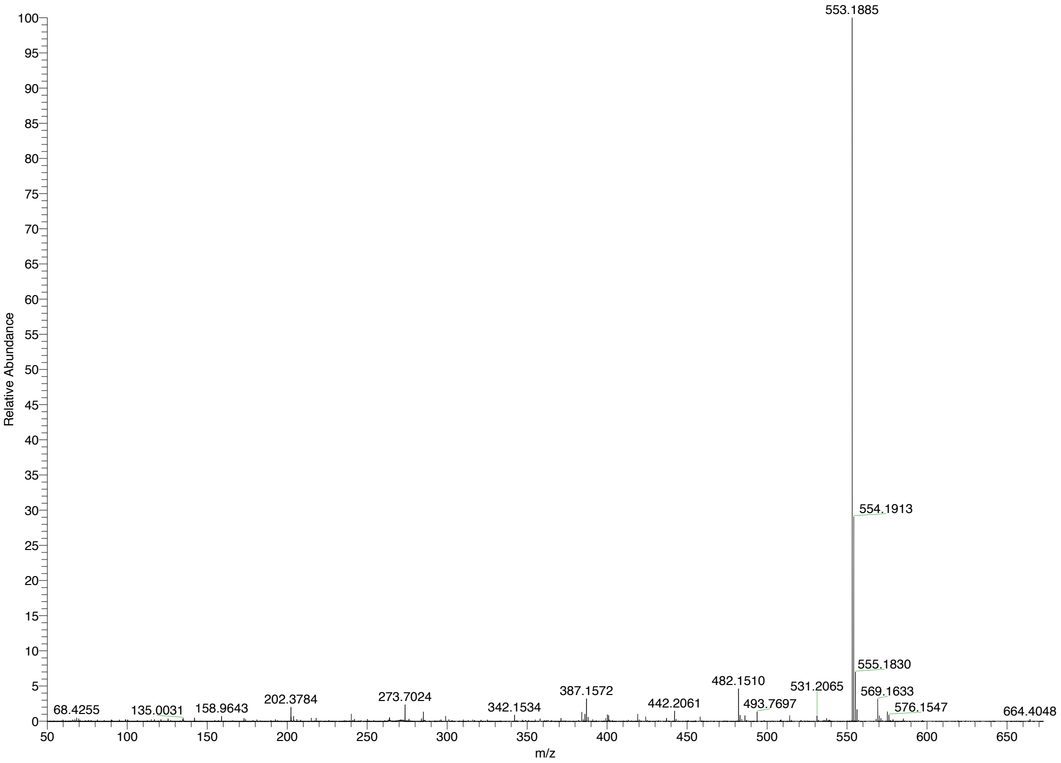

Figure S10. HRMS ESI+ of peptide **I'**.

Peptide II’

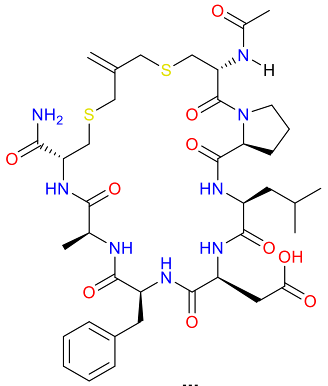

II’

HPLC: Rt = 10.47 min (Grad: water 0.1% TFA/acetonitrile (70:30) → (63.5:36.5), 11 min, λ = 212 nm).
HRMS ESI+ (m/z) calcd. for $C_{35}H_{57}N_8O_{10}S_2[M+H]^+$ 861.3634, found 861.3630.

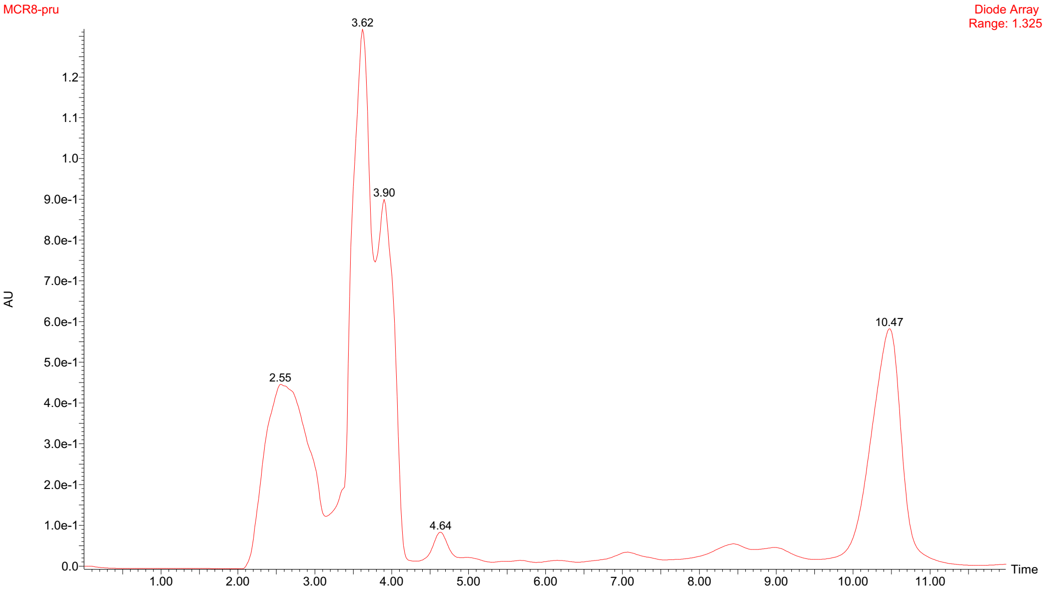

Figure S11. HPLC chromatogram of peptide **II’**.

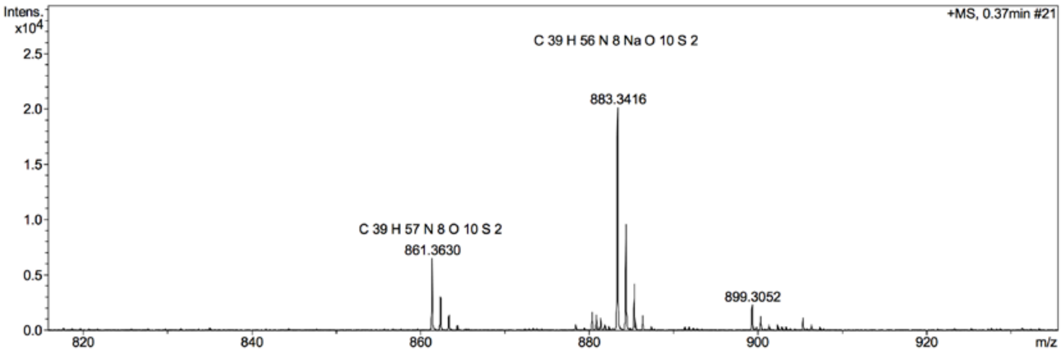

Formula	m/z	z	|err| [ppm]	Mean err [ppm]	mSigma
C 39 H 56 N 8 Na O 10 S 2	883.3453	1+	4.2	3.8	6.0
C 39 H 57 N 8 O 10 S 2	861.3634		0.4	-1.0	6.2

Figure S12. HRMS ESI+ of peptide **II’**.

Peptide III'

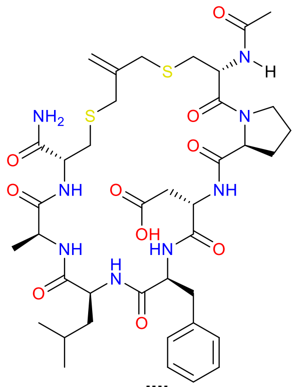

III'

HPLC: Rt = 10.37 min (Grad: water 0.1% TFA/acetonitrile (70:30) → (63.5:36.5), 11 min, λ = 212 nm). HRMS ESI+ (m/z) calcd. for $C_{35}H_{57}N_8O_{10}S_2[M+H]^+$ 861.3634, found 861.3634.

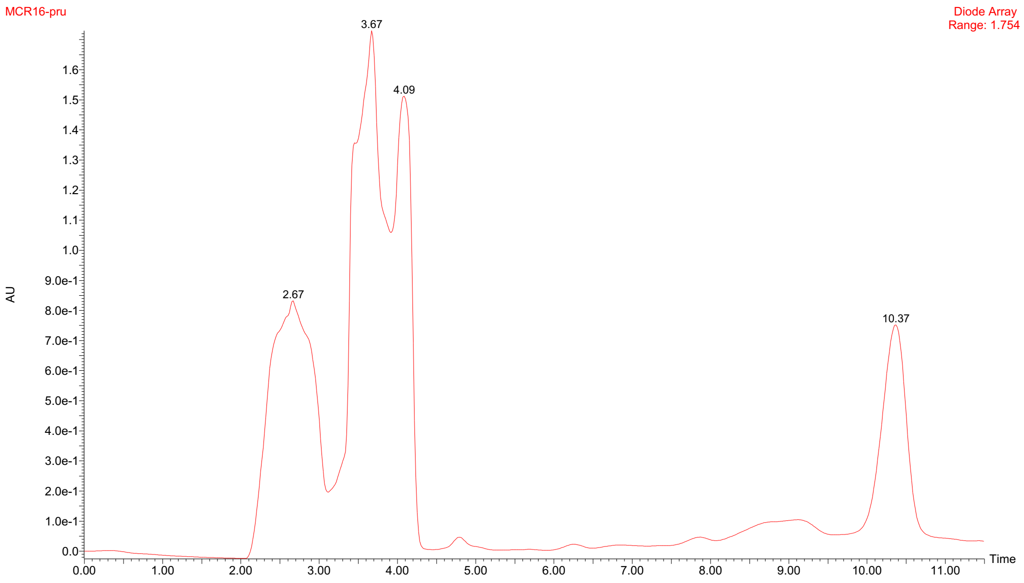

Figure S13. HPLC chromatogram of peptide **III'**.

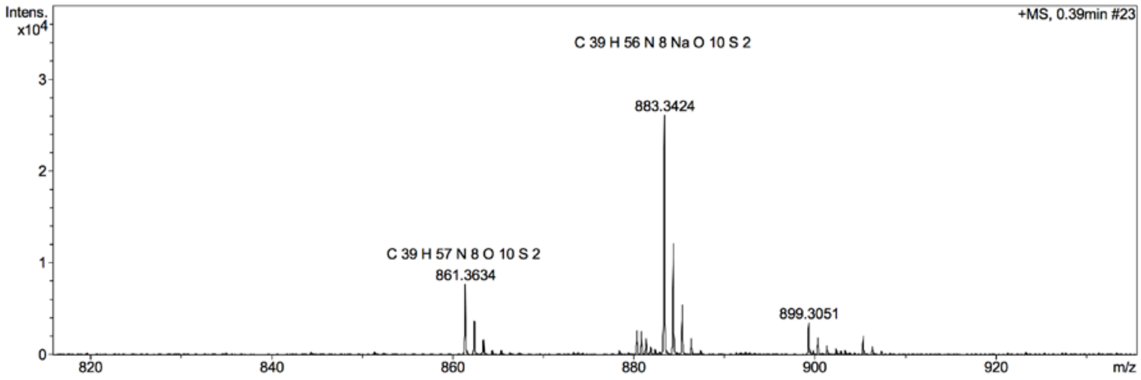

Formula	m/z	z	|err| [ppm]	Mean err [ppm]	mSigma
C 39 H 56 N 8 Na O 10 S 2	883.3453	1+	3.3	3.1	8.4
C 39 H 57 N 8 O 10 S 2	861.3634		0.1	-0.1	24.2

Figure S14. HRMS ESI+ of peptide **III'**.

Peptide IV’

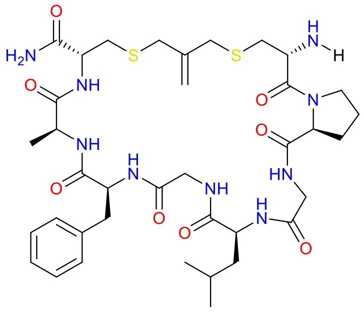

IV’

HPLC: Rt = 8.07 min (Grad: water 0.1% TFA/acetonitrile (70:30) → (63.5:36.5), 11 min, λ = 212 nm).
HRMS ESI+ (m/z) calcd. for $C_{35}H_{58}N_9O_9S_2[M+H]^+$ 860.3793, found 860.3794.

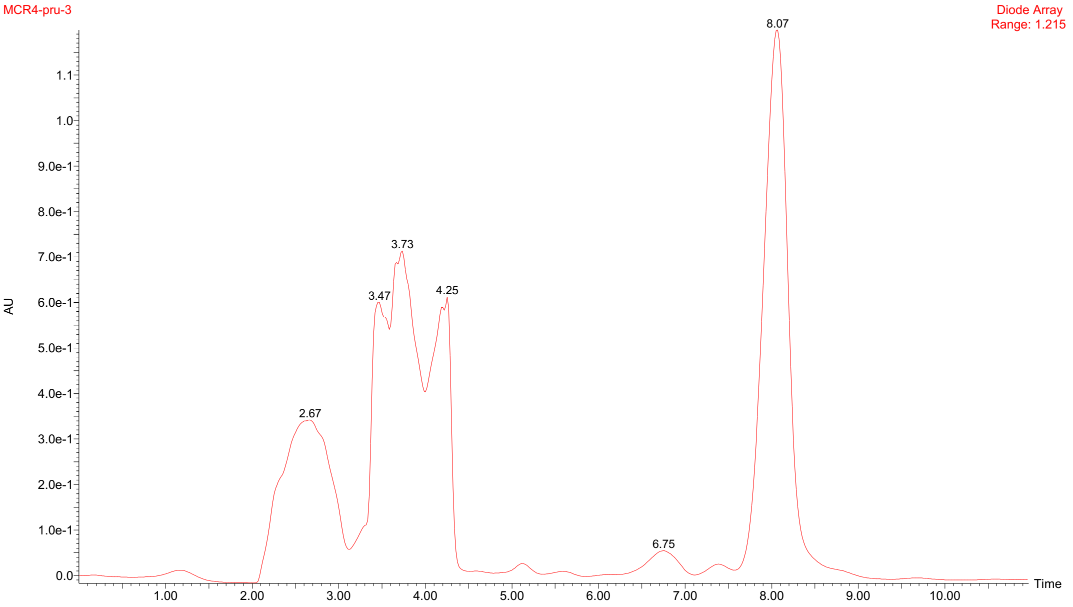

Figure S15. HPLC chromatogram of peptide **IV’**.

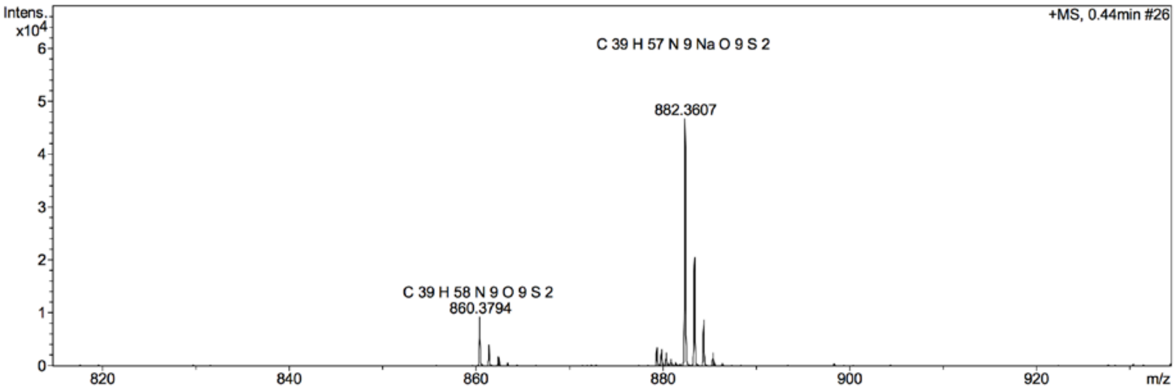

Formula	m/z	z	|err| [ppm]	Mean err [ppm]	mSigma
C 39 H 57 N 9 Na O 9 S 2	882.3613	1+	0.7	0.2	23.2
C 39 H 58 N 9 O 9 S 2	860.3793		0.0	-0.3	17.5

Figure S16. HRMS ESI+ of peptide **IV’**.

Synthesis of stapled Somatostatin

To a solution of 1 mg/mL Somatostatin (6.5 mg, 4.0 μmol, 1.0 equiv) in H_2O was added TCEP (1.7 mg, 6.0 μmol, 1.5 equiv) and K_2CO_3 (1.7 mg, 12.0 μmol, 3.0 equiv). The mixture was stirred at room temperature for 1 h. A solution of 3-bromo-2-bromomethyl-1-propene (1.7 mg, 8.0 μmol, 2.0 equiv) in DMF (0.6 mL) was added and stirred for 24 h at room temperature. After concentrated, the residue was purified by HPLC to obtain the stapled Somatostatin (6.8 mg, 100%) and analysed by LC-MS. Observed mass, 1692; calculated mass $[M+H]^+$, 1692; calculated for somatostatin (1637 Da) + isobutylene (54.05 Da).

HPLC: Rt = 12.80 min (Grad: water 0.1% TFA/acetonitrile (73:27), 15 min, λ = 212 nm). HRMS (ESI+) m/z: calcd. for $C_{80}H_{111}N_{18}O_{19}S_2$ $[M+H]^+$ 1691.7709 found: 1691.7663. Flow rate: 3.5 mL/min.

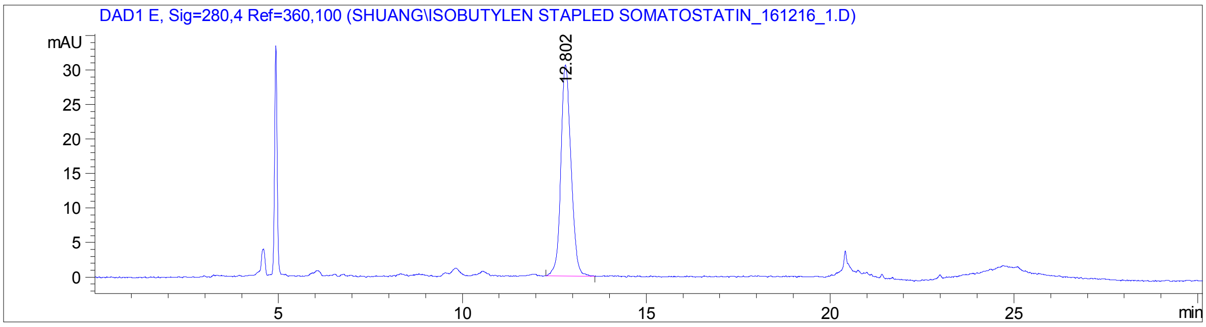

Figure S17. HPLC chromatogram of stapled Somatostatin.

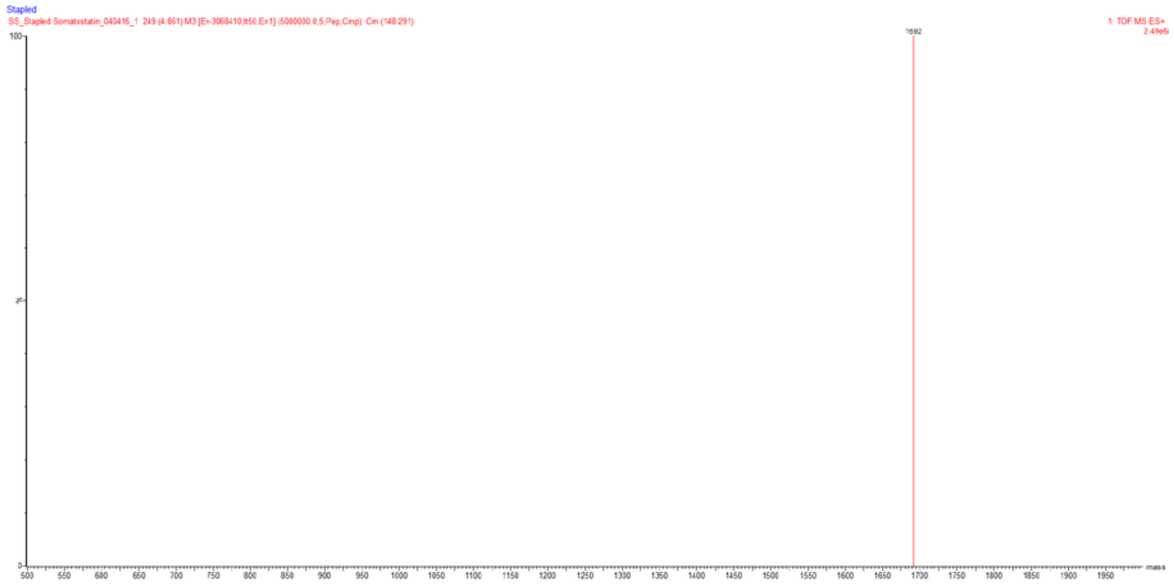

Figure S18. Deconvoluted MS data for stapled Somatostatin.

Circular Dichroism spectroscopy

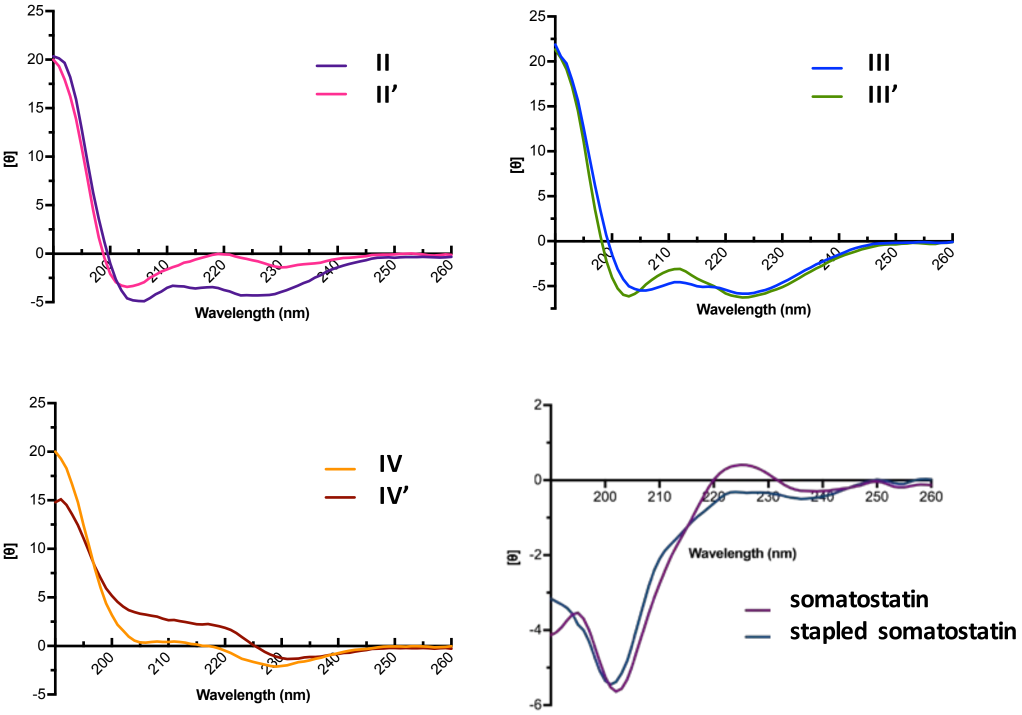

Figure S19. CD spectroscopy of linear and stapled peptides.

Parallel Artificial Membrane Permeability Assay (PAMPA)

Table S1. The Parallel Artificial Membrane Permeability Assay (see General Methods above).

Compound	pH	Avg. Pe	SD Pe	Avg. %R	SD %R	Avg. logPe	SD logPe	Domain, nm
**II**	7.4	7.6	0.8	7	12	-5.12	0.05	245 - 498
**III**	7.4	<0.01						240 - 400
**IV**	7.4	6.0	0.3	1	1	-5.22	0.02	245 - 498
**II'**	7.4	12	6	4	6	-4.96	0.21	245 - 498
**III'**	7.4	10	1	4	6	-4.99	0.02	245 - 498
**IV'**	7.4	13	5	1	1	-4.90	0.18	245 - 498
Ketoprofen	7.4	1.3	0.04	6	1	-5.90	0.01	250 - 360
Verapamil.HCl	7.4	64	4	10	3	-4.20	0.02	250 - 320
Propranolol	7.4	57	3	21	5	-4.25	0.03	250 - 360

P_e - effective permeability ($\times 10^{-6}$ cm/sec) measured directly from assay
pH - refers to the values in donor compartment. Acceptor had a special sink buffer (ASB) at pH 7.4.
%R – membrane retention.
Avg – the value is reported as an average of quadruplicates

Tryptophan fluorescence spectroscopy

Fluorescence spectroscopy was used to determine the dissociation constants of SSTR2 against Somatostatin and Stapled Somatostatin. All experiments were carried out in a Cary Eclipse spectrofluorometer (Varian) at 25 °C with SSTR2 at 1 μM, and concentrations of peptides varying from 0.1 to 7 μM in 25 mM Tris, 150 mM NaCl, pH 7.5 buffer. Fluorescence emission spectra were recorded in the 300-400 nm range with an excitation wavelength of 280 nm, with slit width of 5 nm. The data analysis was performed in Prism (GraphPad software) considering a model with a single binding site.

Stability test of Stapled Somatostatin in presence of GSH and human plasma

A 100 μL aliquot of Stapled Somatostatin (around 1 mM) in H_2O was treated with 5.3 μL of a 20 mM GSH solution (to 1 mM) or 1 μL of reconstituted human plasma (Sigma-Aldrich), and the resulting mixture vortexed for 30 s and then shaken at 37 °C. After 48 h, an aliquot of each reaction mixture was analysed by HPLC. No significant degradation of stapled peptide was observed at 48 h.

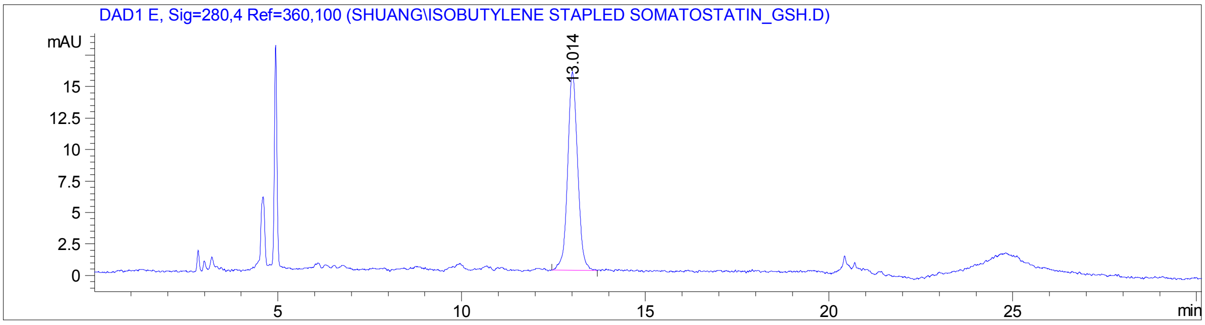

Figure S20. Isobutylene Stapled Somatostatin with GSH for 48 h at 37 °C

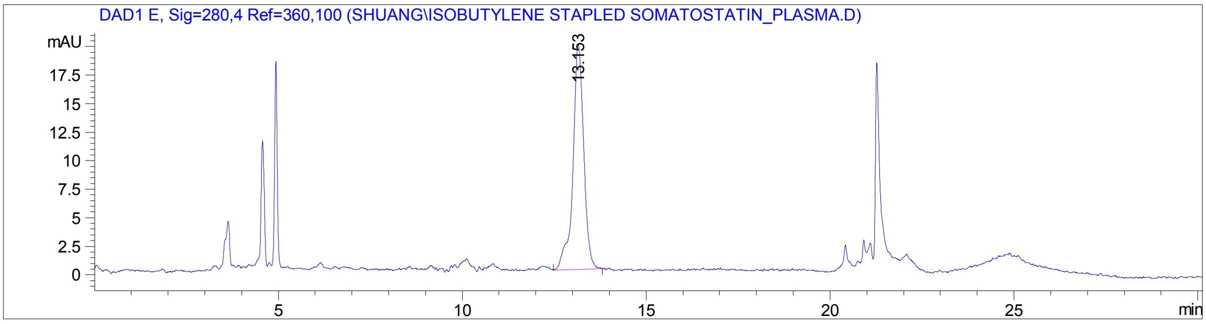

Figure S21. Isobutylene Stapled Somatostatin with plasma for 48 h at 37 °C

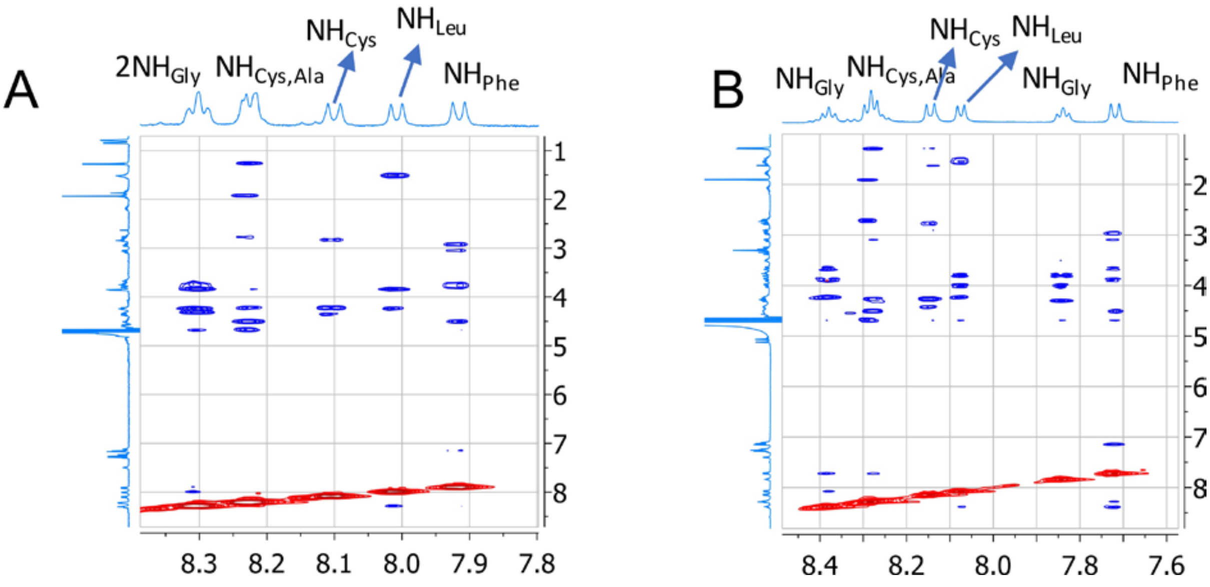

Figure S22. Section of the 500 ms ROESY spectra (400 MHz) of peptides **IV** (A) and **IV'** (B) in H_2O/D_2O (9:1) at 298 K and pH=6.5, showing amide–aliphatic cross-peaks.

Table S2. Comparison of the experimental and MD-tar-derived distances for peptides **IV** and **IV'** in water solution.

peptide **IV**

Distance (Å)	Exp.	MD-tar
$NH_{Leu4} - H\alpha_{Leu4}$	2.5	2.8
$NH_{Phe6} - H\alpha_{Phe6}$	2.5	2.8
$NH_{Cys8} - H\alpha_{Cys8}$	2.6	2.8
$NH_{Ala7} - H\alpha_{Phe6}$	2.0	2.3
$NH_{Cys8} - H\alpha_{Ala7}$	2.1	2.3

peptide **IV'**

Distance (Å)	Exp.	MD-tar
$NH_{Cys1} - H\alpha_{Cys1}$	2.3	2.6
$NH_{Leu4} - H\alpha_{Leu4}$	2.3	2.4
$NH_{Phe6} - H\alpha_{Phe6}$	2.4	2.7
$NH_{Ala7} - H\alpha_{Ala7}$	2.3	2.2
$NH_{Cys8} - H\alpha_{Cys8}$	2.4	2.2

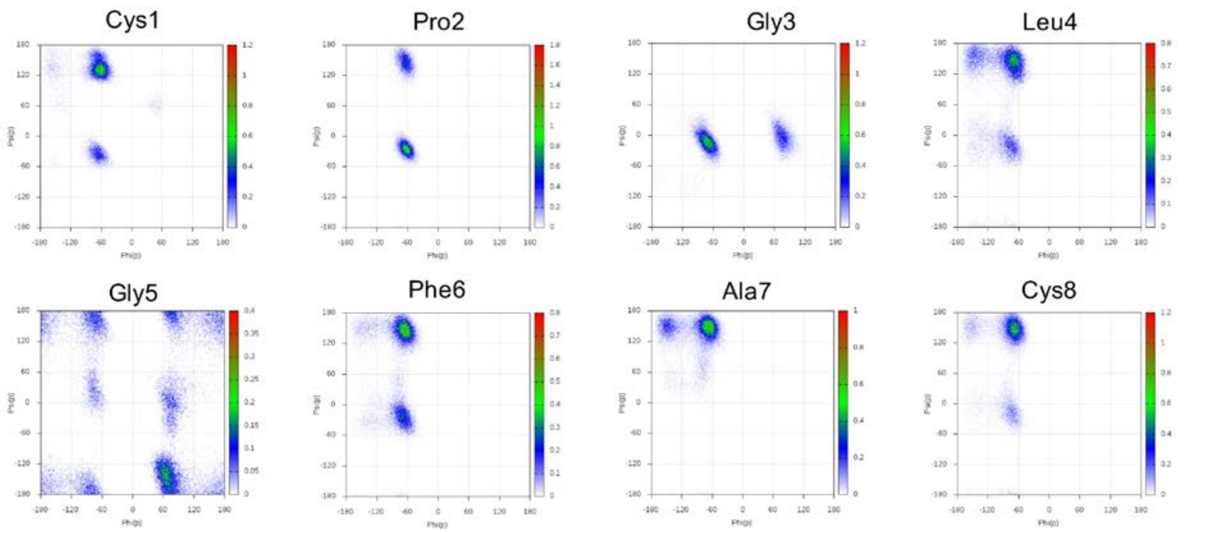

Figure S23. ϕ/ψ distributions obtained by 20 ns MD-tar simulations in explicit water for the peptide backbone of peptide **IV**.

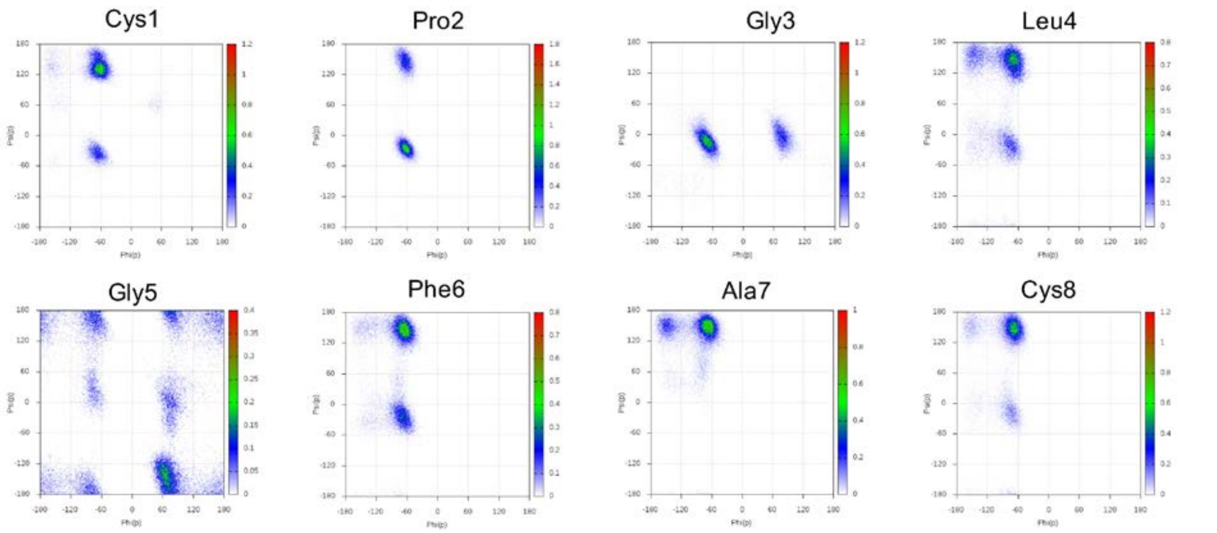

Figure S24. ϕ/ψ distributions obtained by 20 ns MD-tar simulations in explicit water for the peptide backbone of peptide **IV’**.

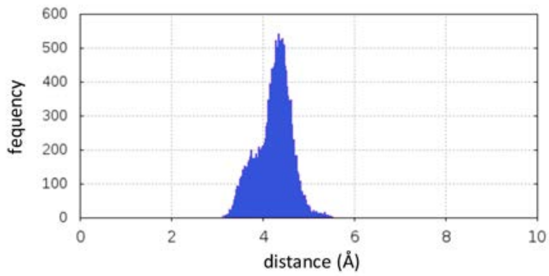

Figure S25. ϕ/ψ Distribution of distance S-S in peptide **IV’** obtained by 20 ns MD-tar simulations in explicit water.

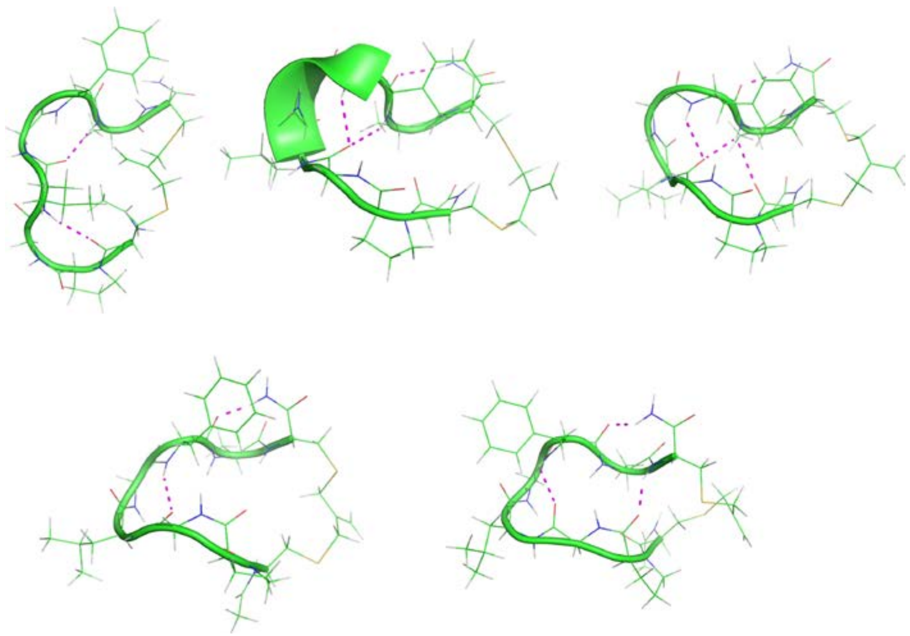

Figure S26. Frames obtained by 20 ns MD-tar simulations in explicit water for peptide **IV',** showing the intramolecular hydrogen bonds (magenta dashed line).

References

[S1] D.A. Case, D.S. Cerutti, T.E. Cheatham, III, T.A. Darden, R.E. Duke, T.J. Giese, H. Gohlke, A.W. Goetz, D. Greene , N. Homeyer, S. Izadi, A. Kovalenko, T.S. Lee, S. LeGrand, P. Li, C. Lin, J. Liu, T. Luchko, R. Luo, D. Mermelstein, K.M. Merz, G. Monard, H. Nguyen, I. Omelyan, A. Onufriev, F. Pan, R. Qi, D.R. Roe, A. Roitberg, C. Sagui, C.L. Simmerling, W.M. Botello-Smith, J. Swails, R.C. Walker, J. Wang, R.M. Wolf, X. Wu, L. Xiao, D.M. York and P.A. Kollman (2017), AMBER 2017, University of California, San Francisco.

[S2] J. A. Maier, C. Martinez, K. Kasavajhala, L. Wickstrom, K. E. Hauser, C. Simmerling, *J. Chem. Theory Comput.* **2015**, *11*, 3696−3713.

[S3] J. Wang, R. M. Wolf, J. W. Caldwell, P. A. Kollman, D. A. Case, *J. Comput. Chem.* **2004**, *25*, 1157−1174.

[S4] A. Jakalian, D. B. Jack, C. I. Bayly, *J. Comput. Chem.* **2002**, *23*, 1623−1641.

[S5] K. Kiyohara, K. Gubbins, A. Panagiotopoulos, *Mol. Phys.* **1998**, *94*, 803−808.

[S6] T. Darden, D. York, L. Pedersen, *J. Chem. Phys.* **1993**, *98*, 10089−10092.

[S7] D. R. Roe, T.E. III. Cheatham, *J. Chem. Theory Comput.* **2013**, *9*, 3084–3095.
